# Peculiarities of the Regulation of Gene Expression in the Ecl18kI Restriction–Modification System

**Published:** 2013

**Authors:** O. Yu. Burenina, E. A. Fedotova, A. Yu. Ryazanova, A. S. Protsenko, M. V. Zakharova, A. S. Karyagina, A. S. Solonin, T. S. Oretskaya, E. A. Kubareva

**Affiliations:** Chemistry Department, Lomonosov Moscow State University, Leninskie Gory, 1, bld. 3, Moscow, Russia, 119991; Belozersky Institute of Physico-Chemical Biology, Lomonosov Moscow State University, Leninskie Gory, 1, bld. 40, Moscow, Russia, 119991; Skryabin Institute of Biochemistry and Physiology of Microorganisms, pr. Nauki, 5, Pushchino, Moscow Region, Russia, 142290; Gamaleya Research Institute of Epidemiology and Microbiology, Gamaleya Str., 18, Moscow, Russia, 123098; Institute of Agricultural Biotechnology, Timiryazevskaya Str. 42, Moscow, Russia, 127550

**Keywords:** restriction–modification systems, (cytosine-5)-DNA methyltransferase, DNA–protein interactions, transcriptional regulation

## Abstract

Transcription regulation in bacterial restriction–modification (R–M) systems is an important process, which provides coordinated expression levels of tandem enzymes, DNA methyltransferase (MTase) and restriction endonuclease (RE) protecting cells against penetration of alien DNA. The present study focuses on (cytosine-5)-DNA methyltransferase Ecl18kI (M.Ecl18kI), which is almost identical to DNA methyltransferase SsoII (M.SsoII) in terms of its structure and properties. Each of these enzymes inhibits expression of the intrinsic gene and activates expression of the corresponding RE gene via binding to the regulatory site in the promoter region of these genes. In the present work, complex formation of M.Ecl18kI and RNA polymerase from* Escherichia сoli *with the promoter regions of the MTase and RE genes is studied. The mechanism of regulation of gene expression in the Ecl18kI R–M system is thoroughly investigated. M.Ecl18kI and RNA polymerase are shown to compete for binding to the promoter region. However, no direct contacts between M.Ecl18kI and RNA polymerase are detected. The properties of M.Ecl18kI and M.SsoII mutants are studied. Amino acid substitutions in the N-terminal region of M.Ecl18kI, which performs the regulatory function, are shown to influence not only M.Ecl18kI capability to interact with the regulatory site and to act as a transcription factor, but also its ability to bind and methylate the substrate DNA. The loss of methylation activity does not prevent MTase from performing its regulatory function and even increases its affinity to the regulatory site. However, the presence of the domain responsible for methylation in the M.Ecl18kI molecule is necessary for M.Ecl18kI to perform its regulatory function.

## INTRODUCTION

Restriction–modification (R–M) systems are abundant in bacterial cells; they contain genes that encode restriction endonucleases (RE ) and DNA methyltransferases (MTases). RE hydrolyzes a certain sequence in a double-stranded DNA (dsDNA), while MTase methylates the same sequence at a strictly determined position, thus preventing its cleavage by RE . The R–M functions as a primitive immune system that protects a host bacterium from penetration by alien DNA: RE hydrolyses the intruding DNA that is not methylated by the corresponding MTase [1]. The activity levels of the RE and the MTase in the cell are to be strictly coordinated. An extremely low level of the MTase gene expression as compared with the RE gene may cause cell death via hydrolysis of cellular DNA, whereas its excessively high level cannot protect the cell against penetration by an alien DNA.

Although there is no doubt that gene expression in the R–M systems is regulated, the mechanisms underlying this process are poorly studied. It has been demonstrated by recent research that coordinated gene expression in R–M systems is presumably determined by regulation at the transcriptional level. Three major types of regulation can be distinguished: via C-proteins, via methylation of the promoter region of the R–M system by the MTase, and via the interaction between the MTase and the regulatory sites in DNA, which differ from the methylation site [2]. This study focuses on the latter type of regulation, which is typical of (cytosine- 5)-DNA MTases (enzymes that methylate the cytosine residue at position 5) belonging to the type II R–M systems. Over 300 (cytosine-5)-DNA MTases have been recently characterized; however, the existence of the regulatory function has been experimentally confirmed only for six of them (M.MspI, M.EcoRII, M.ScrFIA, M1.LlaJI, M.SsoII, and M.Ecl18kI) [2].

The type II R–M system SsoII has been most thoroughly studied. The genes of this system are located in natural plasmid P4 (4250 bp) from the *Shigella sonnei* 47 strain; they are divergently oriented; the intergenic region consists of 109 bp [3]. The other four SsoII-like R–M systems isolated from various bacterial strains have been described; their MTases are either identical to M.SsoII in terms of the amino acid sequence (M.Kpn2kI from *Klebsiella pneumoniae *2k) or differ insignificantly. Thus, MTases Ecl18kI from *Enterobacter cloacae *18k and StyD4I from *Salmonella typhi *D4 carry Met instead of Ile at position 56, while MTase SenPI from *Salmonella enteritidis *P1 contains Ile56 and, in addition, Gly instead of Glu at position 11 [4–7]. The nucleotide sequences of the corresponding genes share 99–100% identity; those of the intergenic regions are absolutely identical. Hence, the data on the functioning of the enzymes from one of these systems can be extrapolated to the other systems as well.

**Fig. 1:**
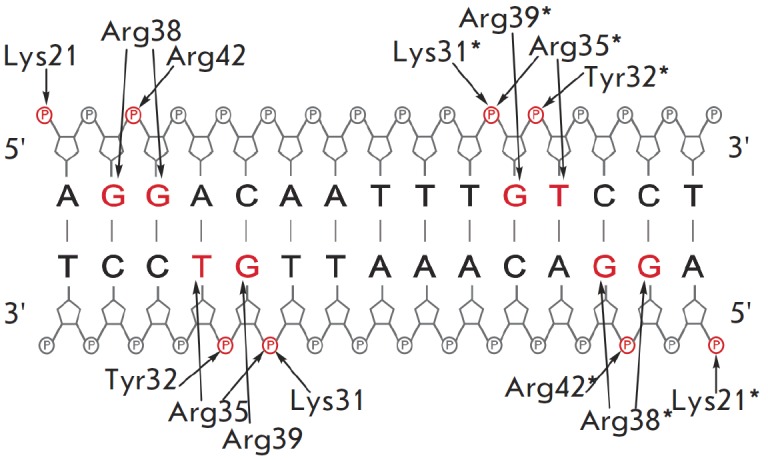
Scheme of the contacts between the amino acid residues in the N-terminal domain of M.SsoII dimer and the regulatory site in DNA. Heterocyclic bases and phosphate groups which interact with M.SsoII (identified by footprinting technique) are shown in red. Amino acid residues of the second M.SsoII subunit are marked with asterisks

All SsoII-like R–M systems recognize sequence 5’-CCN GG-3’/3’-GGNCC -5’ (N = A, G, C or T) in dsDNA and methylate the inner C residue in this sequence in the presence of the cofactor *S*-adenosyl-*L*methionine (AdoMet) forming 5-methyl-2’-deoxycytidine [4]. The promoter elements of the genes encoding the RE and MTase of the SsoII-like R–M systems have been determined using the Ecl18kI system as an example; the *in vitro *transcriptional regulation of these genes by M.Ecl18kI has been also shown. In order to regulate transcription, M.Ecl18kI binds to the so-called regulatory site, the 15-mer inverted repeat 5’-GGACAA**A**TT GTCCT -3’/3’-CCT GTT **T**AACAGGA- 5’, which is localized inside the promoter region of the genes of the Ecl18kI R–M system [9]. The nucleotides that participate in the formation of specific DNA–protein contacts with the MTase are located inside the regulatory site (*Fig. 1*) [10, 11]. All SsoII-like MTases are two-domain proteins whose N-terminal region (residues 1–71) provides transcriptional regulation, while the region of 72–379 residues is responsible for DNA methylation. The N-terminal region of M.SsoII has been shown to have a strongly pronounced secondary structure [12] in which the “helix–turn– helix” (HTH) motif is predicted with a high probability. Two M.SsoII molecules (which are monomeric in the apo-form) interact with the regulatory site [12]. The data [13] regarding the putative contacts in the complex between the M.SsoII N-terminal region and the regulatory site are summarized in *Fig. 1*.

In order to refine the mechanism of gene transcription regulation in the SsoII-like R–M systems, the efficiency of complex formation of M.Ecl18kI and *E. coli* RN A polymerase (RN AP) with DNA fragments containing the regulatory elements of the genes of the Ecl18kI R–M system is assessed in this study. All known SsoII-like R–M systems have been isolated from various enterobacterial strains (*E. coli *belonging to them as well); thus, the use of *E. coli *RN AP is reasonable. The role of residues Lys21, Lys31, Lys46, and Lys53 in the М.Ecl18kI N-terminal region for the binding of this protein to the regulatory site, as well as their effect on the MTase ability to act as a transcription factor and on the interaction between the enzyme and the methylation site are being studied for the first time.

## METHODS

**Protein purification**

MTase Ecl18kI and its mutant forms were purified by affinity chromatography on Ni-NT A agarose [4].* E. coli *RN A polymerase was sequentially purified by Ni-NT A-agarose and heparin-sepharose affinity chromatography, followed by DEAE cellulose ion-exchange chromatography [14].

**Synthesis of DNA fragments I–III**

Fragments **I–III **were synthesized by polymerase chain reaction (PCR ) on an Eppendorf Mastercycler personal thermal cycler (Eppendorf North America, USA). The DNA fragment **I **was obtained using the primers 5’-TT GAGTC ATATGAAGTCTTTCTC -3’ and 5’-AGCAAATGGCGTAATAAAATGC-3’; the DNA fragment **II, **using 5’-TC ATGCATGTCT ACC AGAA-3’ and 5’-CATAAAAAATAACCTTTT ATACT -3’; the DNA fragment **III**, using 5’-TT GAGTC ATATGAAGTC -3’ and 5’-CСAACACTT AATTCT GG-3’. Hybridization (annealing) temperature for each pair of primers was 62, 54, and 46°С, respectively. The PCR cycle (90°С – 60 s, primers annealing – 60 s, 72°С – 40 s) was repeated 25 times. After the PCR , DNA was precipitated with ethanol (2.5 vol.) in the presence of 1 M NaCl. The target DNA was isolated from agarose gel using microcentrifuge tubes Spin-X Centrifuge Tube Filters (Costar, USA).

**Equilibrium binding of the proteins to the DNA ligands**

The 5’-ends of the oligonucleotides were radioactively labeled using T4 polynucleotide kinase (10 units, Fermentas, Lithuania) and [γ^-32Р^]АТP. The complex formation between the М.Ecl18kI and DNA fragments** I–II, **as well as between the RN AP and DNA fragments **I–III, **was conducted in 10 μl of the binding buffer (50 mM Tris-HCl (pH 7.6), 150 mM NaCl, 5 mM β-mercaptoethanol) in the presence of heparin (equimolar amount to the protein) for 40 min at 37°С. In the case of M.Ecl18kI, the reaction mixture contained 1 mM AdoMet. The DNA–protein complex and the unbound DNA duplex were separated by gel electrophoresis in 1% agarose gel. After the electrophoresis, the agarose gels were dried on a supporting plate at 90°С in a hot air flow. The dissociation constants (*K*_d_) of the DNA–protein complexes were determined by the Scatchard technique [15]. The concentrations of М.Ecl18kI and RN AP were 60 and 30 nM, respectively. The concentrations of the DNA duplex** II **were varied within a range from 5 to 120 nM. The complex formation of the mutants М.Ecl18kI(K46A), М.Ecl18kI(K53A), and М.Ecl18kI(K21A) with the DNA fragments **IV **and **V **was conducted in 20 μl of the binding buffer (50 mM Tris-HCl (pH 7.6), 150 mM NaCl, 5 mM DTT , 50 ng/μl poly(dI·dC)) for 20 min at 37°С. The concentrations of the DNA duplexes **IV **and** V **were varied within a range from 20 to 100 nM. The concentrations of М.Ecl18kI(K46A), М.Ecl18kI(K53A), and М.Ecl18kI(K21A) were equal to 560, 400, and 400 nM, respectively, when binding to the DNA fragment** IV **and were equal to 200, 1600, and 5600 nM, respectively, when binding to the DNA fragment **V**.

**Determination of the initial rate of the substrate DNA methylation**

The initial rate of the substrate DNA methylation by MTases Ecl18kI, SsoII, and their mutant forms was determined as previously described [9], on the basis of the degree of the duplex **V **“protection” against hydrolysis by RE Ecl18kI (R.Ecl18kI). For this purpose, 350 nM of the radiolabeled DNA duplex **V **was incubated with MTase in the binding buffer containing 1 mM AdoMet for 0.5–60 min at 37°C. The reaction mixture was then kept at 65°C for 10 min to inactivate the enzyme, and cooled to 25°C. Next, MgCl2 (up to 10 mM) and R.Ecl18kI (up to 240 nM) were added and the reaction mixture was incubated at 37°C for 1 h. The initial active concentrations of the MTases were identical (14 nM). The degree of hydrolysis of the unmethylated DNA duplex** V **by R.Ecl18kI was taken as 100%. The degree of methylation of the DNA duplex **V **by the MTases was calculated with respect to this value, and the kinetic curves were plotted. The initial methylation rate (*v*0) of the DNA duplex **V **by MTase was calculated as an angular coefficient (slope ratio) of the initial linear region on the kinetic curve.

***In vitro *transcription**

The purified DNA fragment **I **(0.25 μg) was incubated with RN AP (3 pmol) in the transcription buffer (40 mM Tris-HCl (pH 7.9), 6 mM MgCl_2_, 10 mM DTT , 10 mM NaCl, 2 mM spermidine) in 8 μl for 10 min at 37°C. Next, the mixture was supplemented with 2 μl of an aqueous heparin solution (0.25 μg/μl) and incubated at 37°C for an additional 10 min. 10 μl of a ribonucleoside triphosphates mixture (UТP (12 μM), АТP, GТР, and СТР (500 μM each)) containing 0.5 μCi [α^-32^P]UТP and 24 units of the RN ase inhibitor RiboLock (Fermentas, Lithuania) were then added. After incubation at 37°C for 1 h, the reaction mixture was mixed with 10 μl of RN A Loading Dye (Fermentas, Lithuania) and loaded onto a polyacrylamide gel.

**Characterization of the regulatory activity of the methyltransferases**

The regulatory activity of the mutant forms of M.Ecl18kI and M.SsoII was assessed via *in vitro *transcription from the DNA fragment **I **in the presence of the corresponding proteins. The wild-type M.Ecl18kI or M.SsoII were used in the control experiments. The reaction mixtures were analyzed by 5% polyacrylamide gel electrophoresis (PAGE; the gel contained 7 M urea) at a field intensity of 5 V/cm in TBE buffer. Only the resulting RN A transcripts contained the radiolabel. In the presence of the SsoII-like MTases capable of acting as regulatory proteins, the following changes were observed: an increase in the radioactivity of the region corresponding to the RN A transcript from the RE gene promoter and a decrease in the radioactivity of the region corresponding to the RN A transcript from the MTase gene promoter. The fraction (%) of the RE gene transcript in the total radioactivity of the resulting transcripts (taken as 100%) at various MTase concentrations was determined. Identical active concentrations of the MTases were used to ensure a correct comparison of the yields of the transcription products in the reaction. They were obtained from the Scatchard plots used to determine the *K*_d_ values for the complexes between the proteins and the duplex **IV **containing the regulatory site [15]. The fraction of the transcript from the RE gene promoter was plotted as a function of the MTase active concentration. The relative yield of this transcript per unit of the MTase active concentration was then determined. For this purpose, the ratio between the angular coefficients (slope ratios) of the initial linear region on the curves of the mutant MTase and the wild-type M.Ecl18kI (or M.SsoII) was calculated.

## RESULTS AND DISCUSSION

**Complex formation of RNA polymerase and M.Ecl18kI with the DNA fragments containing the intergenic region of the Ecl18kI R–M system**

**Fig. 2:**
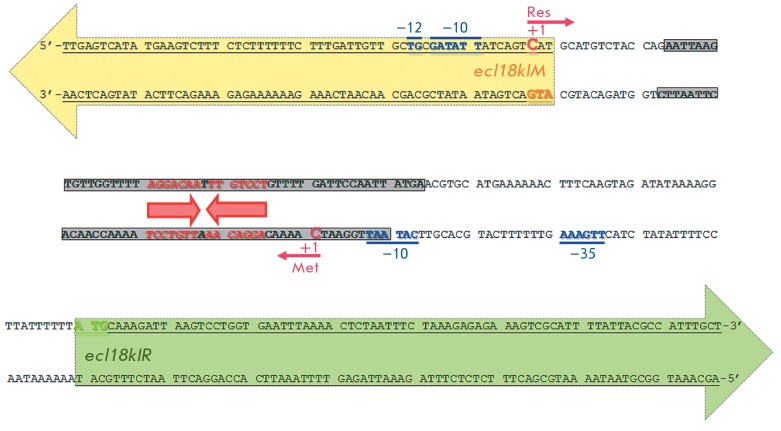
Genetic arrangement of the Ecl18kI restriction–modification system promoter region, the DNA fragment I. The directions of the MTase and the RE genes are shown with yellow and green arrows, respectively. The start codons are also marked with the corresponding colors (yellow or green). The region protected by MTase against DNase hydrolysis is shown in grey. The regulatory site of М.Ecl8kI (inverted repeat) is marked with red letters and red arrows. The transcription initiation points and the promoter elements are shown in pink and blue, respectively

*Figure 2 *shows the genetic arrangement of the Ecl18kI R–M system (based on the data [8, 11]) by the example of the 247-bp DNA fragment **I**. The MTase gene pro moter is localized directly before the regulatory site and partially overlaps the region which is protected by M.Ecl18kI from DNAse I cleavage. We had assumed that the mechanism of negative regulation of the MTase gene expression may consist in physical blocking of the RN AP access to the MTase gene promoter as М.Ecl18kI binds to the regulatory site.

**Fig. 3:**
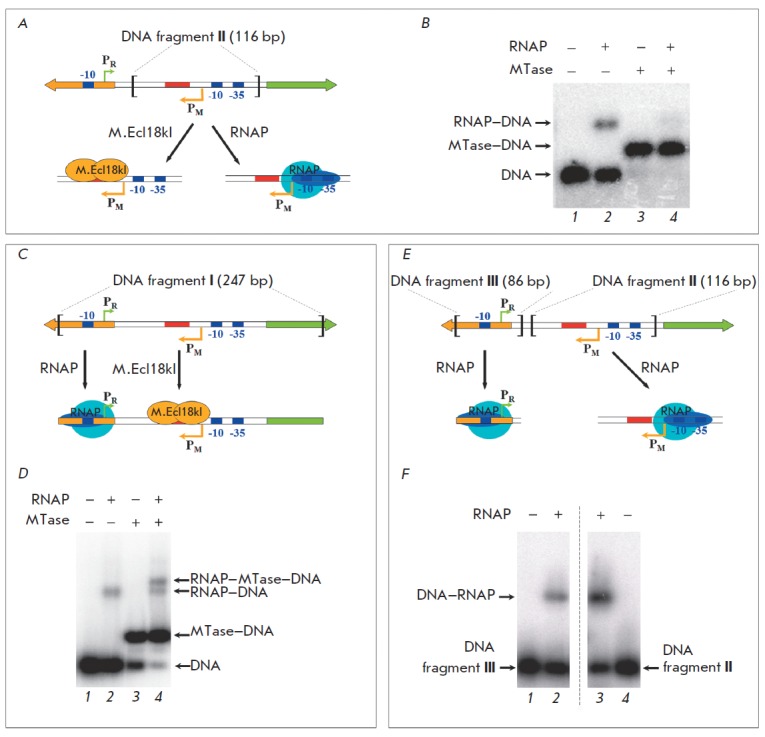
Complex formation of М.Ecl18kI and RNA polymerase with the DNA fragments that contain different elements of the intergenic region of the Ecl18kI restriction–modification system. A, C, E – schematic representations of the DNA– protein complexes formation. The directions of the MTase and the RE genes are shown with yellow and green arrows, respectively. PR, PM – transcription initiation points of the RE and MTase genes, respectively (also marked with thin arrows). The promoter elements are shown in blue, the regulatory site is shown in red. B, D – complex formation of RNA polymerase (30 nM) with the DNA fragments II or I, respectively (15 nM) in the presence or absence of М.Ecl18kI excess (150 nM) under specific binding conditions (with 300 nM heparin). F – complex formation between RNA polymerase (190 nM) and the DNA fragments III or II (30 nM). Radioautographs of 1% agarose gels

To verify this hypothesis, complex formation of both proteins with the 116-bp DNA fragment **II **containing the intergenic sequence of the Ecl18kI R–M system (the regulatory site, the transcription initiation point, and the promoter elements of the MTase gene* ecl18kIM*) but lacking the promoter elements of the RE gene *ecl18kIR *was studied (*Fig. 3A*). After RN AP was added to the MTase–DNA mixture, no other complexes but MTase–DNA and RN AP–DNA emerged in the reaction mixture. This fact eliminates the possibility of direct contact between М.Ecl18kI and RN AP. Moreover, the 5-fold excess of М.Ecl18kI (with respect to RN AP) resulted in virtually complete disappearance of the RN AP–DNA complex. Therefore, MTase binding to the regulatory site does impede the interaction between RN AP and the promoter region of the SsoII R–M system genes (*Fig. 3B*).

The efficiency of RN AP and M.Ecl18kI binding to the MTase promoter and to the regulatory site was assessed by determining the *K*_d_ values of the DNA– protein complexes. *K*_d_ = 12 ± 1 nM for the M.Ecl18kI complex with the DNA fragment **II**, while *K*_d_ = 25 ± 1 nM for the RN AP complex with the same fragment. Thus, the control over the MTase expression level can be attributed to the competition between RN AP and М.Ecl18kI for the binding site. The insignificant (2- fold) difference in the MTase and RN AP affinity to this DNA region allows preventing premature inhibition of M.Ecl18kI synthesis, i.e. controlling the expression level of the MTase gene more accurately. Thus, the level of M.Ecl18kI synthesis does not fall below the minimal value that ensures maintenance of the specific methylation of cellular DNA.

Since the MTase is localized near the transcription initiation point of the RE gene (*Fig. 2*), it seems quite possible that M.Ecl18kI has a negative effect on the *ecl- 18kIR *gene transcription. However, an opposite effect is observed. We had assumed that RN AP and M.Ecl18kI could be bound simultaneously to the same DNA fragment, RN AP interacting with the RE gene promoter, while the MTase interacts with its regulatory site. This assumption is verified experimentally (*Fig. 3C,D*): sequential addition of RN AP and M.Ecl18kI to the 247- bp DNA fragment **I **leads to a ternary complex formation (supposedly RN AP–М.Ecl18kI–DNA) which has a lower electrophoretic mobility as compared with the RN AP–DNA and М.Ecl18kI–DNA complexes. Since two M.SsoII molecules bind to a single regulatory site [12], it is highly probable that each complex (RN AP–М. Ecl18kI–DNA and М.Ecl18kI–DNA) contains two М.Ecl18kI molecules.

Complex formation between RN AP and the two different promoters shows that the degree of RN AP binding to the DNA fragment **III **(*Fig. 3E,F*), which contains the transcription initiation point and the promoter regions of the *ecl18kIR *gene only, is 4-fold lower than the degree of RN AP binding to the DNA fragment **II**, which contains the transcription initiation point and promoter regions of the *ecl18kIM *gene only. Thus, the* ecl18kIM *gene promoter is stronger than the *ecl18kIR* gene promoter, and transcription primarily occurs from the MTase gene promoter in the absence of М.Ecl18kI. This phenomenon can also be stipulated by the “sitting duck” mechanism of transcriptional interference [16] when the rates of the open RN AP complex transition into the elongation form for two closely spaced promoters differ considerably and the activity of the weaker promoter is suppressed due to the intensive transcription of the stronger one.

**Analysis of the ability of the М.Ecl18kI N-terminal region to regulate gene transcription in the restriction–modification system** *in vitro*

The experiments with deletion mutants have demonstrated that the M.SsoII ability to act as a transcription factor can be attributed to the N-terminal region of this protein, which consists of 71 residues [3]. The amino acid sequences of the N-terminal regions of M.Ecl18kI and M.SsoII significantly resemble C-proteins. When comparing the M.SsoII regulatory site with the idealized sequence of C-boxes (5’-GACT ...AGTC -3’) [17], 6 out of 8 nucleotides coincide. Considering the significant variability among the sequences of the C-boxes, the regulatory site recognized by M.Ecl18kI can also be classified as a C-box. The deletion mutant Δ(72–379) M.Ecl18kI, which is the N-terminal region of M.Ecl18kI, retains its strongly pronounced secondary structure and is capable of specific binding to the DNA containing the regulatory site; however, the efficiency of such binding is an order of magnitude lower than that of the full-length protein [12].

**Fig. 4:**
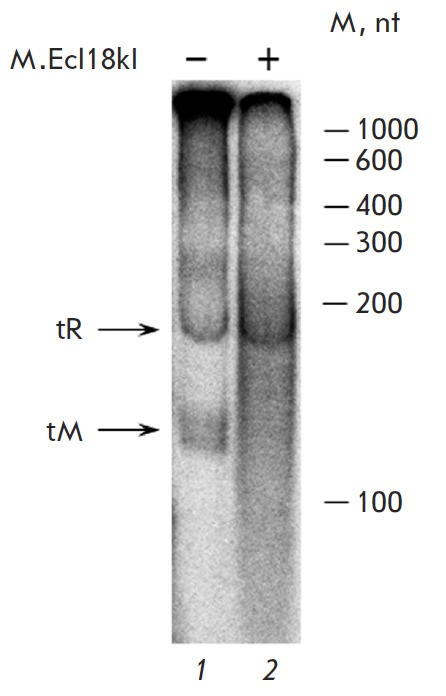
Analysis of the transcripts from the DNA fragment I by electrophoresis in 5% polyacrylamide gel under denaturing conditions. Radioautography: 1 – the transcripts in the absence of M.Ecl18kI, 2 – the transcripts in the presence of M.Ecl18kI 4-fold excess (considering active concentrations of the enzymes). The positions of RNA length markers are shown on the right side. tR – the transcript from the ecl18kIR gene promoter; tM – the transcript from the ecl18kIM gene promoter; nt – nucleotides

**Fig. 5:**
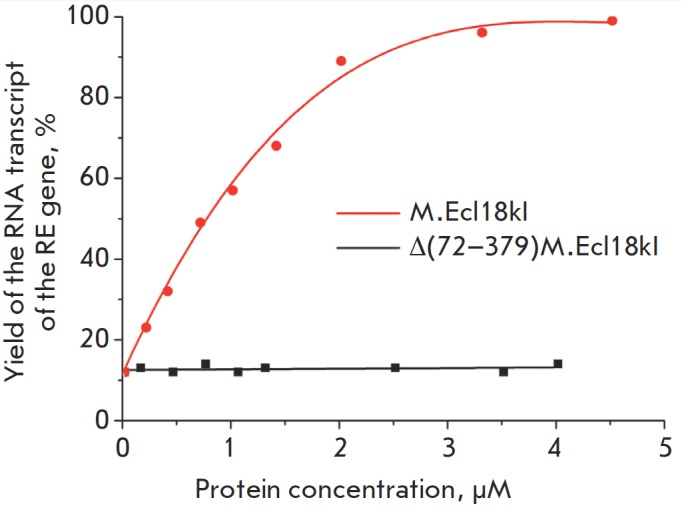
Dependence of the transcript yield from the ecl- 18kIR gene promoter on the concentration of M.Ecl18kI or its deletion mutant Δ(72–379)M.Ecl18kI

The effect of Δ(72–379)M.Ecl18kI on the *in vitro *transcription of the *ecl18kIR *and *ecl18kIM *genes has been studied. The full-length M.Ecl18kI was used in the control experiment (*Fig. 4*). Transcription from the 247-bp DNA fragment **I** resulted in two products corresponding to the transcripts from the RE gene promoter (~190 nucleotides) and from the MTase gene promoter (~110 nucleotides). When the reaction mixture was titrated with increasing amounts of М.Ecl18kI, the fraction of the MTase gene transcript decreased considerably, while that of the RE gene transcript increased (*Figs. 4,5*). Meanwhile, the addition of Δ(72–379)M.Ecl18kI to the reaction mixture caused no changes in the ratio between the yields of the two transcripts; i.e., this deletion mutant could not function as a transcription factor (*Fig. 5*). This fact is probably due to the low affinity of Δ(72–379)M.Ecl18kI to the DNA carrying the regulatory site [12]: such a protein cannot efficiently compete with RN AP for binding to the promoter region. It is also possible that the deletion mutant covers a considerably smaller DNA fragment as compared with the full-length M.Ecl18kI and therefore is not a steric impediment for RN AP. Thus, the region responsible for methylation is necessary to maintain the regulatory function of M.Ecl18kI. This result agrees with the recently proposed structural model of the complex between the SsoII-like MTases and the regulatory site within the intergenic region of the R–M system: the N-terminal regions of both MTase molecules specifically interact with the regulatory site, while the regions responsible for methylation are nonspecifically bound to the DNA flanking the regulatory site [18].

**Model of gene transcription regulation in the Ecl18kI restriction–modification system**

**Fig. 6:**
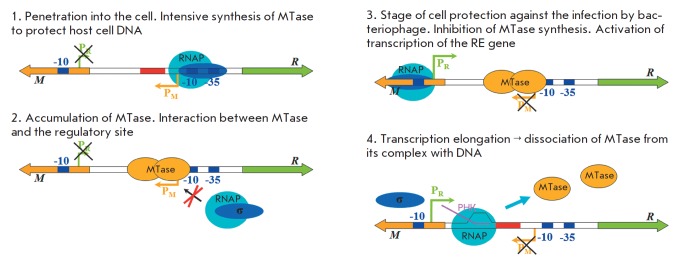
The supposed mechanism of regulation of gene transcription in SsoII-like restriction–modification systems. The designations are similar to those given in the legends to Figs. 2 and 3

After the R–M system penetrates into a cell, the MTase is actively synthesized from the stronger promoter, which is required to protect cellular DNA against the hydrolysis by RE . A certain amount of MTase, which can efficiently protect the cell against bacteriophage infection, is produced with time. Then, two MTase molecules bind to the regulatory site and block the RN AP access to the promoter of the MTase gene (*Fig. 6*). No complex formation between MTase and RN AP occurs in this case; i.e., the mechanism of transcription suppression of the MTase gene is based exclusively on the competition between the MTase and RN AP for binding to the intergenic region of the Ecl18kI R–M system. The close *K*d values attest to the fact that even small changes in the MTase concentration are expected to affect the efficiency of the MTase gene transcription.

The interaction between the M.SsoII regions responsible for methylation with the DNA flanking the regulatory site described in [18] seems to confer additional strength to the DNA–protein complex. This circumstance allows SsoII-like MTase to successfully compete with RN AP for binding to the promoter region, resulting in the suppression of the MTase gene transcription and stabilization of the MTase concentration in the cell. It can be assumed that binding of the enzyme region responsible for methylation to the DNA flanking the regulatory site is a compensatory mechanism which is required to make the effect on transcription of a MTase dimer bound to the regulatory site as efficient as that of two C-protein dimers bound to two palindromic sites in DNA. The fact that a deletion mutant, which is the N-terminal region of M.Ecl18kI, does not have this “additional” interaction explains the low stability of its complex with the DNA and its inability to control transcription in the Ecl18kI R–M system.

Binding between M.Ecl18kI and the regulatory site results in indirect activation of the RE gene promoter by preventing RN AP from binding to the MTase gene promoter. During transcription from the RE gene promoter, RN AP runs against the MTase region, which is responsible for methylation and nonspecifically interacts with the DNA region flanking the regulatory site [18]. These nonspecific DNA–protein contacts can be relatively easily destroyed by RN AP, which melts DNA in the elongation complex. It is possible that both MTase subunits are pushed away from the DNA, which can be caused by the reduced affinity of the enzyme to the DNA melted during the elongation process.

**Effect of single amino acid substitutions on the regulatory activity of the SsoIIlike methyltransferases**

*The mutant form of M.SsoII containing Cys142 substitution in the region responsible for methylation.* Cys142 in the М.Ecl18kI (M.SsoII) molecule plays the key role in catalyzing the methyl group transfer from the reaction cofactor AdoMet to the substrate DNA [19]. Replacement of Cys142 by Ala results in loss of M.SsoII enzymatic activity. The efficiency of the mutant protein binding to the methylation site decreases; however, the mutant has a considerably higher affinity to the regulatory site (*Table*) [9]. The M.SsoII(C142A) ability to regulate *in vitro *transcription of the genes in the Ecl18kI R–M system was tested in this study.

**Fig. 7:**
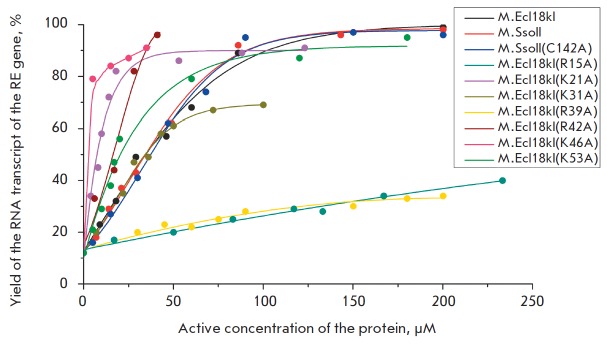
Dependence of the transcript yield from the ecl18kIR gene promoter on the active concentration of M.Ecl18kI, M.SsoII, or the following mutants: M.SsoII(C142A), M.Ecl18kI(R15A), M.Ecl18kI(K21A), M.Ecl18kI(K31A), M.Ecl18kI(R39A), M.Ecl18kI(R42A), M.Ecl18kI(K46A), and M.Ecl18kI(K53A)

The yields of the transcripts of the *ecl18kIR *gene in the presence of M.SsoII, M.Ecl18kI, or the mutant protein M.SsoII(C142A) are almost identical (*Fig. 7, Table*). Hence, loss of the methylation function does not affect MTase’s ability to function as a transcription factor.

*The mutant forms of M.Ecl18kI containing substitutions in the region responsible for the regulatory function.* Based on the model of the complex between the M.SsoII N-terminal region and the regulatory site [13], a hypothesis has been proposed that residues Lys21, Lys31, Arg35, Arg38, Arg39, and Arg42 interact with DNA (*Fig. 1*). We studied the regulatory properties of the М.Ecl18kI mutants, where one of the abovementioned residues was replaced by Ala (*Table*). The М.Ecl18kI mutants with one of the residues (Arg15, Lys46, or Lys53) replaced by Ala were used as a control. The regulatory activity of all the M.Ecl18kI mutant forms was tested by conducting *in vitro *transcription in the presence of these proteins. Wild-type M.Ecl18kI was used in the control experiment.

It is shown for the first time that the amino acid substitutions in the N-terminal region affect the MTase ability to regulate transcription in the R–M system (*Table*). An interesting and unexpected result of the study is the dynamics of the yield changes of the transcripts from the RE gene promoter, which differed among different M.Ecl18kI mutants at the same active concentrations. The mutants exhibiting high affinity to the regulatory site had been expected to regulate transcription more efficiently, whereas the regulation of transcription by the MTases exhibiting lower affinity had been expected to weaken. Indeed, in the presence of M.Ecl18kI(R35A) and M.Ecl18kI(R38A), which poorly interact with the regulatory site (*Table*, [9]), the result is identical to that observed in the absence of the protein: the RN A transcript from the MTase gene promoter is predominant in the reaction mixture. Evidently, these mutants cannot regulate gene transcription in the Ecl18kI R–M system.

**Table:** Characterization of the DNA-binding, regulatory, and methylating activities of the MTases Ecl18kI, SsoII, and their mutant forms^1^

MTases	Relative yield of the RE gene transcript per unit of active concentration of MTase	K_d_ of the complex between MTase and the regulatory site, nM^1,2^	K_d_ of the complex between MTase and the methylation site, nM^1,3^	Relative initial methylation rate^1,3^
Ecl18kI	1.0	224 ± 24	87 ± 12	1
SsoII	1.0	248 ± 33	144 ± 14	1
SsoII(C142A)	1.0	35 ± 3	172 ± 10	-
Ecl18kI(R15A)	0.4	56 ± 13	103 ± 24	< 1
Ecl18kI(K21A)	3.9	48 ± 9	87 ± 3	38
cl18kI(K31A)	1.0	198 ± 29	26 ± 3	29
Ecl18kI(R35A)	-	> 4000	140 ± 12	2
Ecl18kI(R38A)	-	> 4000	96 ± 13	11
Ecl18kI(R39A)	0.4	93 ± 14	266 ± 4	22
Ecl18kI(R42A)	2.5	32 ± 2	256 ± 4	< 1
Ecl18kI(K46A)	13.5	250 ± 32	> 4000	-
Ecl18kI(K53A)	1.8	206 ± 7	> 4000	-

^1^ Data for M.Ecl18kl, M.SsoII, M.SsoII(C142A), M.Ecl18kl(R15A), M.Ecl18kl(R35A), M.Ecl18kl(R38A), M.Ecl18kl(R39A), and M.Ecl18kl(R42A) have been published earlier [9].

^2^ The complex formation was studied using the 31-bp DNA duplex **IV **containing the regulatory site: 5’-TTGGTTTT**AGGACAA*T*TTGTCCT**GTTTTGAT-3’ 3’-AACCAAAA**TCCTGTT*A*AACAGGA**CAAAACGA-5’ (DNA duplex **IV**).

^3^ The complex formation and methylation activity were studied using the 30-bp DNA duplex **V **containing the methylation site: 5’-GATGCTGCCAA**CCTGG**CTCTAGGTTCATAC-3’ 3’-CTACGACGGTT**GGACC**GAGATCGAAGTATG-5’ (DNA duplex **V**).

In the presence of the rest of the M.Ecl18kI mutants, which could bind efficiently to the regulatory site, changes in the amount of the RN A transcript from the RE gene promoter were observed (*Fig. 7, Table*). However, a certain correlation between the MTase affinity to the regulatory site and the transcript yield per unit of the MTase active concentration was detected only for three mutants: M.Ecl18kI(K21A), M.Ecl18kI(K31A), and M.Ecl18kI(R42A) (in addition to the aforementioned M.Ecl18kI(R35A) and M.Ecl18kI(R38A)). In the case of M.Ecl18kI(K21A) and M.Ecl18kI(R42A), the affinity to the regulatory site and the efficiency of transcriptional regulation per unit of the MTase active concentration is higher than that in the case of wildtype M.Ecl18kI. The affinity of M.Ecl18kI(K31A) to the regulatory site and the yield of the RN A transcript per unit of the MTase active concentration is comparable to the same values for the wild-type M.Ecl18kI.

Meanwhile, the mutants M.Ecl18kI(R39A) and M.Ecl18kI(R15A), which are characterized by a higher affinity to the regulatory site as compared with that of wild-type M.Ecl18kI (2.5- and 4-fold, respectively), regulate transcription in the Ecl18kI R–M system less efficiently. The RN A transcript yield per unit of active concentration for M.Ecl18kI(K46A) and M.Ecl18kI(K53A) is 13- and 1.8-fold higher than that for the wild-type M.Ecl18kI, respectively, despite the fact that the dissociation constants of their complexes with the 31-bp duplex** IV **containing the regulatory site are comparable.

The absence of a correlation between the affinity to the regulatory site and the RN A transcripts yield can be attributed to the fact that the *K*_d_ value shows the thermodynamic stability of the MTase–DNA complex, while the relative yield of the transcription product per unit of the MTase active concentration characterizes the rate of the MTase–DNA complex formation indirectly.

**Methylation of the substrate DNA**

We have studied the impact onto the M.Ecl18kI methylation function caused by the replacement of residues Lys21, Lys31, Lys46, or Lys53 in the M.Ecl18kI N-terminal region by Ala. For this purpose, the *K*_d_ values of the complexes between the MTase mutants and the 30- bp duplex **V **containing the methylated site, together with the methylation rate of this substrate, were determined (*Table*). The mutants M.Ecl18kI(K21A) and M.Ecl18kI(K31A) efficiently bind to the substrate duplex **V**. The rate of DNA methylation by these proteins is 30- to 40-fold higher as compared to that of the wild-type enzyme. Contrariwise, the mutants M.Ecl18kI(K46A) and M.Ecl18kI(K53A) are characterized by low affinity to the duplex **V **and cannot methylate it.

Similar studies were conducted for the mutant proteins where one of the Arg residues (R15, R35, R38, R39, or R42) located in the M.Ecl18kI N-terminal region was replaced by Ala (*Table*) [9]. The following conclusions can be drawn when comparing the results. The affinity of M.Ecl18kI mutants to the substrate DNA **V **generally decreases when the amino acid substitution approaches the M.Ecl18kI region responsible for methylation (*Table*). Thus, the *K*d values of the M.Ecl18kI(R15A), M.Ecl18kI(K21A), and M.Ecl18kI(R38A) complexes with the duplex **V **coincide within the experimental error with those for the wild-type M.Ecl18kI. The M.Ecl18kI(K31A) affinity to the DNA duplex **V **is even 3-fold higher. In M.Ecl18kI(R35A), the affinity to the methylation region is 1.6-fold lower than in the wildtype MTase; in M.Ecl18kI(R39A) and M.Ecl18kI(R42A), it is 3-fold lower; whereas M.Ecl18kI(K46A) and M.Ecl18kI(K53A) virtually do not bind to the duplex** V**.

M.Ecl18kI(K21A), M.Ecl18kI(K31A), and M.Ecl18kI(R39A) methylate the substrate duplex **V** very efficiently; however, no correlation in the affinity to the methylation site or in the transcription regulation capacity is observed among these mutant forms (*Table*). A higher degree of the duplex **V **methylation (as compared with that of M.Ecl18kI) was also observed for M.Ecl18kI(R38A) with the regulatory function turned off. The efficiency of M.Ecl18kI(R35A) methylation remains virtually unchanged, although its affinity to the methylation site decreases 1.6-fold when the regulatory function is turned off. The replacement of Arg15 and Arg42 by Ala results in a 2.5- to 3-fold decrease in the enzyme’s methylation ability. The mutants M.Ecl18kI(K46A) and M.Ecl18kI(K53A) cannot methylate the duplex **V **because of their extremely low affinity.

Thus, our results demonstrate that amino acid substitutions in the M.Ecl18kI region responsible for regulation affect the ability of this protein to bind to the substrate DNA and methylate it, although no clear pattern is observed among the mutant forms.

## CONCLUSIONS

The modification enzyme (cytosine-5)-DNA MTase Ecl18kI *in vitro *regulates transcription of the genes in the Ecl18kI restriction–modification system. The inhibition of the MTase gene transcription is caused by competition between RN AP and the modification enzyme for the binding site near the MTase gene promoter. Transcription of the restriction endonuclease Ecl18kI gene is activated due to the attenuation of transcriptional interference resulting from the modification enzyme binding to the regulatory site. It is demonstrated for the first time that the presence of the MTase region responsible for methylation is required for this enzyme to function as a transcription factor. The point mutation turning off the MTase catalytic function increases the mutant affinity to the regulatory sequence and does not affect its ability to act as a transcription factor. On the other hand, the mutants M.Ecl18kI(K46A) and M.Ecl18kI(K53A), which efficiently regulate transcription in the Ecl18kI R–M system, do not modify the substrate DNA because of the extremely low affinity to the methylation site. The replacement of Arg35 or Arg38 in MTase Ecl18kI by Ala not only impairs protein binding to the regulatory site, but also impedes its performing of the regulatory function; however, the efficiency of DNA methylation is considerably enhanced in this case. Evidently, there is a relationship between the functioning of the two DNA recognition centers in the SsoII-like MTases.

## Abbreviations

MTase: DNA methyltransferase
PAGE: polyacrylamide gel electrophoresis
RNAP: RNA polymerase
R–М system: restriction–modification system
RE: restriction endonuclease
AdoMet: S-adenosyl-L-methionine
М.Ecl18kI: DNA methyltransferase Ecl18kI
М.SsoII: DNA methyltransferase SsoII
R.Ecl18kI: restriction endonuclease Ecl18kI. Prefix “d” for designating deoxyribonucleosides, oligodeoxyribonucleotides, and DNA duplexes is omitted
